# Haematopoiesis in Zebrafish (Danio Rerio)

**DOI:** 10.3389/fimmu.2022.902941

**Published:** 2022-06-02

**Authors:** Michał Stosik, Beata Tokarz-Deptuła, Wiesław Deptuła

**Affiliations:** ^1^Institute of Biological Science, Faculty of Biological Sciences, University of Zielona Góra, Zielona Góra, Poland; ^2^Institute of Biology, University of Szczecin, Szczecin, Poland; ^3^Institute of Veterinary Medicine, Faculty of Biological and Veterinary Sciences, Nicolaus Copernicus University in Toruń, Toruń, Poland

**Keywords:** haematopoietic stem cells, embryonic period haematopoiesis, post-embryonic haematopoiesis, haematopoietic niches, *Danio rerio (zebrafish)*

## Abstract

Haematopoiesis in fish and mammals is a complex process, and many aspects regarding its model and the differentiation of haematopoietic stem cells (HSCs) still remain enigmatic despite advanced studies. The effects of microenvironmental factors or HSCs niche and signalling pathways on haematopoiesis are also unclear. This review presents *Danio rerio* as a model organism for studies on haematopoiesis in vertebrates and discusses the development of this process during the embryonic period and in adult fish. It describes the role of the microenvironment of the haematopoietic process in regulating the formation and function of HSCs/HSPCs (hematopoietic stem/progenitor cells) and highlights facts and research areas important for haematopoiesis in fish and mammals.

## Introduction

Haematopoiesis in vertebrates, despite many studies, remains to be fully characterised, and there are even controversies related to the model of this process and its details, particularly the differentiation of haematopoietic stem cells (HSCs) (1). As emphasized by Cheng et al. (1), these controversial observations might be related to differences in research techniques, especially methods for tracing the development/differentiation of HSCs and labelling efficiency of these cells. Haematopoiesis is still enigmatic, and perhaps this is the key issue, as a process strongly dependent on various factors of the microenvironment and signalling pathways that influence all stages of the formation and development of blood cells and their precursors (2). In the light of previous findings that confirmed the hierarchy of haematopoietic stem cells and hematopoietic stem/progenitor cells (HSPCs), as well as the differentiation models of HSCs (3–5), Cheng et al. (1), proposed new paradigms on haematopoietic stem cell differentiation, emphasizing at the same time the need for their further revision (6–10). In the proposed model (1) it is assumed that HSCs differentiate into multipotent progenitors (MPP), which include the subpopulation of short-term haematopoietic stem cells (MPP1/ST-HSC), giving rise to parallel subpopulations: MPP2, MPP3 and MPP4 (LMPP, lymphoid-primed multipotent progenitors). At the further stages of differentiation, the MPP2 subpopulation gives rise to pre-megakaryocyte-erythrocytes and ultimately the platelet lineage, with the indirect participation of the megakaryocyte progenitors and the erythrocyte lineage, mediated by the pre-colony forming unit-erythroid. The MPP3 subpopulation gives rise to granulocytes and monocytes that form from pre-granulocyte-macrophages and granulocyte-macrophage progenitors, and the MPP4 subpopulation (LMPP) differentiates, through common lymphoid progenitors mainly into lymphocytes. It should be stressed that the long-term research into details of haematopoiesis has provided many new data, especially on the differentiation of HSCs, giving reasons and foundations for the modification or even radical change of the model/paradigm of haematopoiesis (1). However, studies indicating that haematopoiesis may be characterised by the continuous acquisition of specific properties by hematopoietic stem/progenitor cells (HSCs/HSPCs) seem particularly interesting, since these cells are likely to have an epigenetic status allowing for the transformation towards a specific cell lineage or specific cell type (1, 11–15). Yokota (14) indicates the possible heterogeneity of HSCs and describes the process that corresponds to the “holacracy”. Xu et al. (16), by visualizing haematopoiesis as a stochastic process, showed that the formation of blood cells can be modelled as a dynamic process with a stochastic competition between the cell types. Haematopoiesis might also be a process based on deterministic events, as can be inferred from the study by Zhen et al. (17) in *Danio rerio*. Studies on zebrafish (*D. rerio*) also imply that haematopoiesis may be a continuous process of HSPCs’ differentiation associated with the simultaneous suppressive and/or stimulating transcriptional activity of genes that are responsible for the formation, proliferation and differentiation of cells specific for a particular haematopoietic lineage (18). *Danio rerio* (Kingdom – *Animalia*, Superphylum – *Deuterostomia*, Phylum – *Chordata*, Subphylum – *Vertebrata*, Class – *Actinopterygii*, Order – *Cypriniformes* Family – *Cyprinidae*, Subfamily – *Danioninae*, Genus – *Danio*, Species - *D*. *rerio*) due to its special biological characteristics is a model organism and a highly valuable and effective “tool” in studies on haematopoiesis and haematopoietic niches in vertebrates, including mammals and humans (19–22). Findings from studies on the zebrafish model have explained processes that influence the course of haematopoiesis and the development of HSCs, and have significant implications not only for general knowledge in the range of basic sciences, but most of all are important because of their potential applicability in regenerative medicine (2, 18, 19, 21–23).

## *Danio Rerio* as a Model Organism in Studies on Haematopoiesis

Developmental processes and molecular mechanisms regulating haematopoiesis in embryos and larvae of *D*. *rerio*, as emphasized by Gore et al. (2), are conserved in evolutionarily younger organisms. It is also very important that the zebrafish has cells of all the haematopoietic lineages, available in each period of differentiation in the pronephros, which is the equivalent to mammalian bone marrow, and orthologs of many transcription factors (TFs), including TAL bHLH transcription factor 1 (TAL1, erythroid differentiation factor), GATA binding protein 2 (GATA2), RUNX family transcription factor 1 (RUNX1), MYB proto-oncogene, transcription factor (MYB, known as c-myb), and ETS transcription factor ERG (ERG), which play important roles in the process of haematopoiesis in mammals (24–30). The essential similarity between the haematopoiesis in zebrafish *D*. *rerio* and mammals also includes transcription mechanisms, more specifically the transcriptional status of cells, associated with the expression of genes coding regulatory factors, crucial for a specific cell lineage, as well as signalling pathways important for the regulation of haematopoiesis, including the Wnt signalling pathway and Notch signalling pathway (2, 18, 23, 31–33). It is noteworthy that Notch signaling targeting the transcription factor RUNX1 controls self-renewal of stem cells, and the Notch-Runx1 signaling pathway is essential for the fate of these cells (33). *D*. *rerio* is characterised by easy and fast reproduction (zebrafish is oviparous and fertilization is an external process); dynamic development (at 25-26°C); embryonic transparency, which enables observations and *in vivo* imaging of the development of embryos/larvae and haematopoiesis; ease of genetic testing and genetic modification to generate transgenic organisms using the Tol2 Transposase system (autonomous transposone identified in Japanese rice fish, *Oryzias latipes*, which is used to create the transgenic zebrafish) to obtain reporter lines suitable for specific labelling of certain types of cells with green fluorescent protein (GFP) (2, 18–20, 34–39). A model enabling the visualization of, inter alia, the hematopoietic process is *Danio rerio* double mutant - the *nacre* mutant and the spontaneous mutant the *roy orbison* (*roy*), known as the *casper* strain, which shows a complete lack of melanocytes and iridophores in embryogenesis and in adulthood. These fish retain the transparency of the outer shells throughout their lives and, very importantly, they are a tool with the expected sensitivity and resolution in imaging and analyzing the number and distribution of GFP-labeled stem cells *in vivo* (40–42). In zebrafish all events of blood cell formation and colonization of haematopoietic niches can be observed from the earliest stages of development (from a few hours and/or days after fertilization), at the single cell level. The effects of modified/downregulated expression of a specific gene (gene knockdown) or its removal or permanent deactivation (gene knockout) caused by the use of an antisense oligonucleotide (morpholino oligonucleotides) that binds to the coding gene or its mRNA, the CRISPR system and endonuclease Cas9 (CRISPR/Cas9) or transcription activator-like effector nucleases (TALEN) can also be analysed successfully in zebrafish (2, 19, 36). However, the analysis of the expression of a specific gene cannot ignore the fact that there are significant differences between phenotypes caused by genetic mutations and phenotypes caused by gene knockdown or knockout, also in *D*. *rerio*. It should also be borne in mind that harmful phenotype changes in mutants, but not in morphants, may be buffered by the activity of the mechanism underlying genetic compensation (43, 44). Therefore, conclusions reached from studies on haematopoiesis in zebrafish can be and are indeed used for modelling studies on haematopoiesis and its disorders in humans (20, 32). Nevertheless, it should be noted that despite the large research opportunities offered by model organisms of *D*. *rerio*, which complement the mammalian models, they are biased with certain limitations, which was indicated by Konantz et al. (20). For example, there is a limited availability of antibodies suitable for labelling cell surface markers or techniques for the simultaneous and selective expression of oncogenes in the tissues of adult *D*. *rerio*, which would offer more opportunities for the phenocopying of human disorders.

## Embryonic Haematopoiesis in *Danio Rerio*

**Figure 1:**
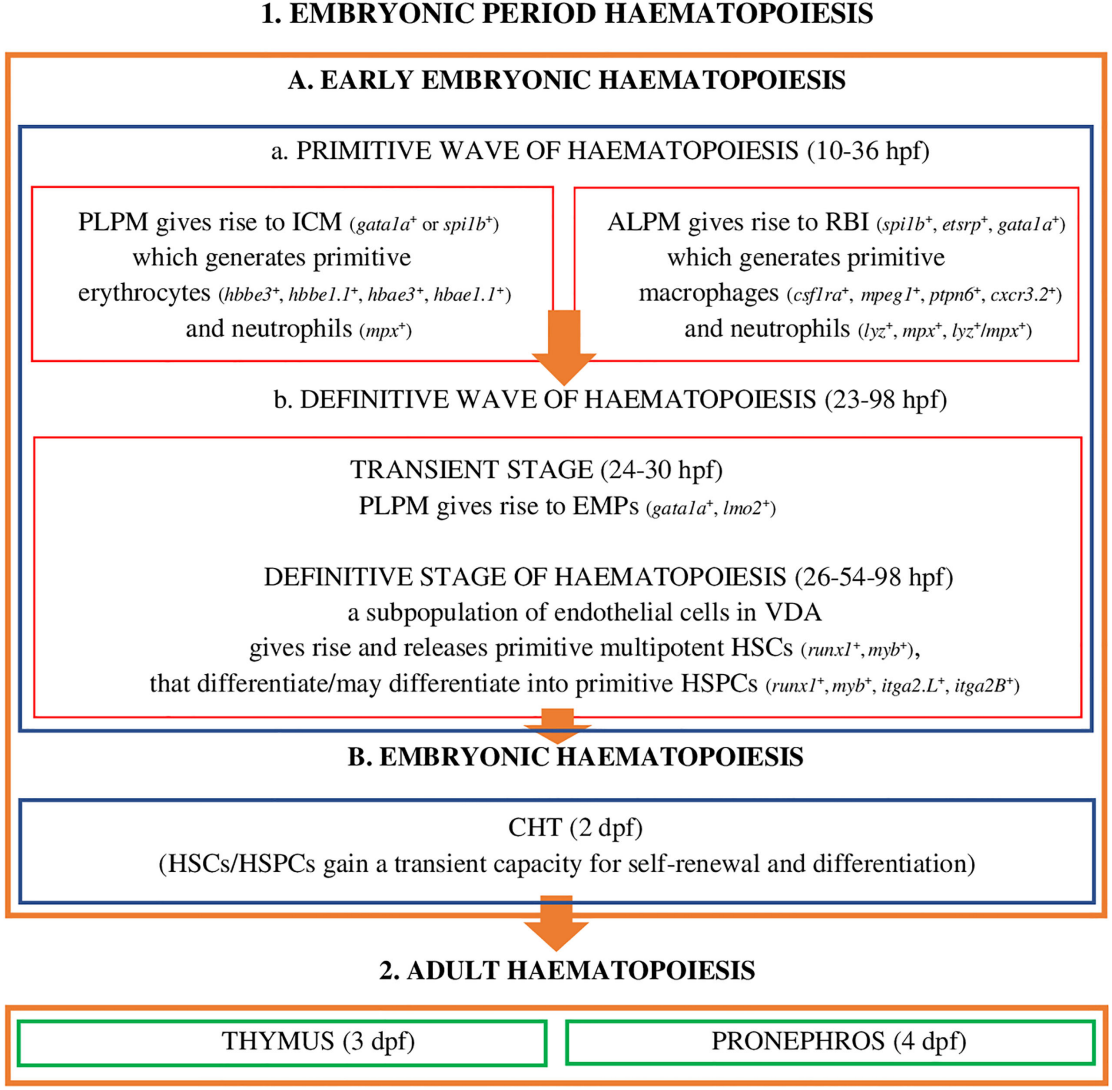
Embryonic period haematopoiesis and adult haematopoiesis in zebrafish (D. rerio) (references in the main text). Explanatory notes for part **(A, B)**: PLPM, posterior lateral-plane mesoderm; ALPM, anterior lateral-plane mesoderm; ICM, intermediate cell mass; RBI, rostral blood islands; VDA, ventral wall of the dorsal aorta; CHT, caudal hematopoietic tissue; hpf, hours post fertilization; dpf, days after fertilization; spi1b, Spi-1 proto-oncogene b; etsrp, ETS1-related protein; gata1a, GATA binding protein 1a; hbbe3, haemoglobin beta embryonic-3, hbbe1.1, haemoglobin beta embryonic-1.1; hbae3, haemoglobin alpha embryonic-3, hbae1.1, haemoglobin alpha embryonic-1.1, lyz, lysozyme C, mpx, myeloid-specific peroxidase, lzm/mpx, double-positive neutrophils stain strongly with Sudan black; lmo2, LIM domain only 2; runx1, RUNX family transciption factor 1; myb, MYB proto-oncogene, transcription factor; itga2.L, integrin subunit alpha 2 L homeolog (itga2-A); itga2B, integrin subunit alpha 2 B (CD41).

Embryonic period haematopoiesis in *D*. *rerio* (**Figure 1**) has two stages, i.e. early-embryonic haematopoiesis (**Figure 1.1A**) and embryonic haematopoiesis (**Figure 1.1B**).

### Early-Embryonic Haematopoiesis

At this stage of embryonic development, hematopoiesis in zebrafish (**Figure 1.1A**) is a process which, as in other vertebrates, develops in two successive waves: the primitive wave (**Figure 1.1A.a**) and the definitive wave (**Figure 1.1A.b**) (2, 20, 23, 45).

The primitive wave of the early-embryonic haematopoiesis (**Figure 1.1A.a**) starts in two different regions of the lateral mesoderm: in the posterior lateral-plate mesoderm (PLPM) and in the anterior lateral-plate mesoderm (ALPM). From PLPM, at the trunk midline of the fish embryo (2), the so-called intermediate cell mass (ICM) blood islands are formed (gata1a+ - GATA binding protein 1a or spi1b+ - Spi-1 proto-oncogene b, called pu.1) conceptually analogous to the extra - embryonic yolk sac blood islands of mammals and birds, which give rise to primitive erythroid cells - erythrocytes (E) (hbbe3+ - haemoglobin beta embryonic-3, hbbe1.1+ - haemoglobin beta embryonic-1.1, hbae3+ - haemoglobin alpha embryonic-3, hbae1.1+ - haemoglobin, alpha embryonic 1.1, known as hbae1), and 24 hours post fertilization (hpf) they enter the circulation as oval and nucleated cells and primitive myeloid cells - neutrophils (mpx+ - myeloid-specific peroxidase) (2, 19, 23, 28). Moreover, transcription factors Gata1a and Spi-1b exhibit a cross-inhibitory relationship to regulate the fates of primitive erythroid and myeloid cells (23). ALPM, which is the main myelopoietic site, gives rise to rostral blood islands (RBI) (*etsrp^+^* - ETS1-related protein, *spi1b^+^*, *gata1a^+^*), where primitive macrophages are formed (*csf1ra^+^* - colony stimulating factor 1 receptor a, *mpeg1*^+^ - macrophage expressed gene 1, *ptpn6^+^* - protein tyrosine phosphatase non-receptor type 6, *cxcr3.2^+^* - chemokine [C-X-C motif] receptor 3, tandem duplicate 2) as well as neutrophils (*lyz^+^* - lysozyme C, *mpx^+^*, *lyz^+^/mpx^+^* - double-positive neutrophils stain strongly with Sudan Black), which have a phagocytic capacity, and are involved in the formation of HSCs/HSPCs in the ventral wall of the dorsal aorta (VDA) and the migration of these cells to the vascular system/venous sinuses in the caudal region of the fish body, between the caudal artery and the vena cava, i.e. to the caudal haematopoietic tissue (CHT) (2, 19, 23, 46–53). As highlighted by Jagannathan-Bogdan and Zon (23), PLPM and ALPM in *D. rerio* co-expressing *tal1*, *gata2*, *lmo2* (LIM domain only 2), *fli1* (Fli-1 proto-oncogene, ETS transcription factor) and *etsrp* may give rise to angioblasts or HSCs, which confirms the presence of haemangioblasts - common precursors of endothelial cells and haematopoietic cells in zebrafish and humans.

The definitive wave of early-embryonic haematopoiesis (**Figure 1 - 1.Ab**) includes the transient stage and the definitive stage of haematopoiesis. The transient stage takes place in PLPM, where multipotent erythromyeloid progenitors (24-30 hpf) are formed, also called erythroid-myeloid progenitors (*gata1a^+^*, *lmo2^+^*) (2, 19, 23, 54, 55). During the definitive stage of haematopoiesis the subpopulation of endothelial cells in VDA (the equivalent of the mammalian aorta-gonad-mesonephros [AGM] region), in the process of the so-called endothelial-hematopoietic transformation (EHT), also defined as the new type of cell behaviour, primitive multipotent HSCs (runx1+, myb+) are formed and released (26-54 hpf) that may differentiate into haematopoietic stem progenitor cells (HSPCs) expressing runx1, myb, itga2.L (integrin subunit alpha 2 L homeolog, known as itga2-A), itga2.B (integrin subunit alpha 2 B, known as CD41) (2, 4, 18, 19, 21, 47, 56, 57). According to Henninger et al. (58), about 30 HSCs or their clones are generated at this stage of embryonic development, and these cells are responsible for the formation of the haematopoietic system and life-long haematopoiesis in fish/vertebrates. Henninger et al. (58) also emphasized that HSCs in *D*. *rerio* in this period of embryonic development are the most productive. This event, i.e. the formation of HSCs/HSPCs, marks the beginning of the definitive haematopoiesis, in which blood cells are generated by self-renewal and differentiation of already existing HSCs/HSPCs but not those generated *de novo* (19, 58). HSCs/HSPCs are induced, for example, by Cxcl12b (chemokine [C-X-C motif] ligand 12b) produced by the specific population of endothelial precursors (endotome cells), and by TNF-α (tumour necrosis factor α), produced by primitive macrophages and neutrophils, and after moving into the vena cava they migrate to CHT (2 days post fertilization, dpf), which is the site of embryonic haematopoiesis (the equivalent of the foetal liver in mammals).

### Embryonic Haematopoiesis

In CHT they are stimulated by cytokines: Kitlgb (kit ligand b), Osm (oncostatin M), Csf3a (colony stimulating factor 3 [granulocyte] a), Ccl25b (chemokine [C-C motif] ligand 25b), Cxcl8b (chemokine [C-X-C motif] ligand 8b) and Cxcl12a (chemokine [C-X-C motif] ligand 12a) as well as Klf6a (Krüppel-like factor 6a, transcription factor), and gain the capacity for self-renewal and differentiation. The development and expansion of these cells is a key property of CHT, supervised, inter alia, by the regulatory mechanism of non-hematopoietic CHT components, i.e. vascular endothelial cells, epithelial cells, fibroblasts, and nerve and muscle cells (59–61). It has been shown (59) that promoting the proliferation and differentiation of HSCs/HSPCs in the CHT niche, in addition to chemokines and cytokines, is also tightly controlled by various external and internal cellular factors, such as the cell cycle and transcriptomic features that are likely to affect cell heterogeneity in the parental and progenitor compartments. An example is the identified and characterized (59) vascular endothelial-specific factor, i.e. Gpr182 (G protein-coupled receptor 182), which plays a positive role in CHT remodeling favoring the expansion of HSPCs. It should be emphasized that the HSPCs population, in CHT *D*. *rerio*, includes four subpopulations, identified on the basis of different lineage-specific genes - HSPC1 *cmyb*^+^, a proliferative subpopulation not involved in the differentiation process and HSPC2 *hemgn*^+^, *tmem14ca*^+^, HSPC3 *cebpb*^+^, HSPC4 *coro1a*^+^, *ccr9a*^+^, *rac2*^+^, subpopulations capable of differentiating cells targeted to perform specialized functions (59). These subpopulations are also recognizable on the basis of the metabolic genes HSPC2 *pcna*^+^, *cdk1*^+^, *slc11a2*^+^, HSPC3 *fosab*^+^, HSPC4 *actb1*^+^, *rac2*^+^, *litaf*^+^, *coro1a*^+^ (59), suggesting their influence on HSPC heterogeneity (59). At this stage of development of hematopoiesis, HSCs/HSPCs give rise to embryonic macrophages, neutrophils and monocytes, they proliferate and migrate (19, 49, 56, 59–62) and finally colonize the developing thymus, where T lymphocytes are generated (3 dpf), along with the pronephros (4 dpf) (the equivalent to the bone marrow of mammals) (**Figure 1. - 2**) (2, 19, 32, 63, 64). In the pronephros, HSCs/HSPCs fulfil their life-long haematopoietic function (19).

### Haematopoietic Niches

The key sites in regulating the formation and function of stem and progenitor cells, HSCs/HSPCs, are specialized/specific anatomical regions called haematopoietic niches creating a microenvironment of the haematopoietic process. They have special anatomical and structural features, that is a specific subset of cells including vascular endothelial cells, mesenchymal stromal cells (MSC), macrophages and neutrophils, and regulatory agents that interact with stem cells and selectively orchestrate a development pathway for these cells (19, 36). Two haematopoietic niches have been identified in *D*. *rerio*: VDA, called the initiating haemopoietic niche, and CHT, defined as the primary tissue of embryonic haemopoiesis or the intermediate haematopoietic niche. In adult zebrafish, the haematopoietic niche is the pronephros, which accommodates self-renewing and differentiating HSCs/HSPCs, and generating all blood cells during the whole adult life of vertebrates (19, 28, 36).

Some vascular endothelial cells in VDA, called haemogenic endothelial cells (HE) in *D*. *rerio* give rise to HSCs (*runx1*^+^, *myb*^+^) and HSPCs (*runx1*^+^, *myb*^+^, *itga2.L*^+^, *itga2B*^+^), while others perform regulatory functions in the process of their formation. The formation of HSCs/HSPCs, as previously mentioned, is induced by the activity of a specific population of endothelial precursors, called “endotome cells” (at the primary stage of their formation in somite they are identified as *meox1*^+^, mesenchyme homeobox 1), which migrate and colonize VDA (65), and Cxcl12b produced by them. Further, due to the deactivation of *meox1* (a gene coding a protein which may play a role in the molecular signalling network regulating the growth of somites) and increasing the number of “endotome cells”, the induction of HSCs/HSPCs is stronger and they are released to the circulation, with the involvement of cells other than endothelial cells from VDA, i.e. those from adjacent somites, as well as primitive macrophages and neutrophils by the release of cytokines and enzymes, such as TNFα or Mmp2 (matrix metalloproteinases 2 (19, 36, 47, 51, 58). Monteiro et al. (66) demonstrated that the formation of HSCs/HSPCs is also regulated by Tgf-β (transforming growth factor beta), an anti-inflammatory cytokine regulating the proliferation and differentiation of many types of cells. Tgf-β, by binding to a single type II serine-threonine kinase receptor (Tgf-βr2) in an autocrine mechanism activated by ligands, Tgf-β1a and Tgf-β1b, is involved in the determining/programming of endothelial cells towards the haemogenic endothelium (before EHT), and further, in a paracrine mechanism activated by Tgf-β3 (the source of the ligand is the notochord), it is involved in EHT and the generation of HSCs/HSPCs. Tgf-β, in both stages of its activity, also regulates the expression of Jag1a (jagged canonical Notch ligand 1a) activating the Notch1a receptor present on the surface of cells in VDA, which is a necessary process for the formation of haematopoietic stem cells (66, 67). The release of HSCs/HSPCs from VDA is regulated by Cbfβ (core binding factor subunit beta), coded by the *cbfβ* gene whose expression defines HE cells and determines EHT (68). A study by Bresciani et al. (68) revealed, however, the involvement and functional role of Cbfβ at different stages of HSCs/HSPCs development. Cbfβ is a subunit involved in the early stage of the definitive wave of haematopoiesis (**Figure 1.1.A.b**) by forming the Runx1/Cbfβ complex, which, as in mammals (mice), is a heterodimeric core-binding transcription factor) (68). In *D. rerio* both subunits, Cbfβ and Runx1, can be active independently of each other and participate in two different stages of the definitive haematopoietic wave (68). Runx1 is involved in the generation of HSCs (*runx1*^+^, *myb*^+^) in VDA, while Cbfβ is involved in the release of HSCs from VDA. The definitive wave of haematopoiesis in *D. rerio* is also regulated by two isoforms of the Scl transcription factor (stem cell leukaemia) - Sclβ and Sclα (17). These isoforms are involved in the defining and generation of HE – Sclβ (VDA *scl-β^+^* cells, before EHT) and the generation, retention and release of HSCs/HSPCs - Sclα (generated HSCs *scl-α^+^*) (17). Of note is the fact that the expression of *scl-β* is the first molecular marker of haemogenic endothelial cells (17), and the process of HSCs generation is determined by a sequential activity of transcription factors: Scl-β, Runx1 (at the stage of EHT) and Scl-α. At this stage of haematopoiesis the differentiation of haematopoietic stem cells HSCs/HSPCs is also strongly regulated by Dnmt3bb.1 (DNA [cytosine-5-]-methyltransferase 3 beta, duplicate b.1), an enzyme that is one of six (dnmt3bb.1, dnmt3aa, dnmt3ab, dnmt3ba, dnmt3bb.2, dnmt3bb.3) homologs of DNMT3b in mammals (69, 70). The activity of Dnmt3bb.1 initiated in response to an increased expression of the *runx1* gene (stimulated most likely by Scl-β (17),) and the Notch/Runx1 signalling pathway, sustains the expression of the *myb* gene and functional efficiency of HSCs/HSPCs (69, 70). Moreover, the vascular endothelial cells present in haematopoietic niches support the process of haematopoiesis regardless of their origin (VDA, CHT, pronephros) and different transcriptional characteristics (32, 71, 72). Importantly, the endothelial cells in the CHT are characterized by a specific alignment, for which HSCs/HSPCs migrating and released to the perivascular space must squeeze between them. Because of this, haematopoietic stem cells directly surrounding endothelial cells induce changes in their organization within the niche (a pocket is formed around HSCs/HSPCs) and probably also cause an increase in the concentration of cytokines and signalling molecules, which consequently influences the expansion and differentiation of HSCs/HSPCs and their effective interaction with stromal cells (19, 22, 36).

Mesenchymal stromal cells (MSCs) in *D*. *rerio* originate from the ventral border of the caudal somites and are generated during the epithelial–mesenchymal transition (EMT). MSCs are present among the endothelial cells of vascular network/vascular sinuses forming CHT and may express Cxcl12a cytokines (19, 32, 73). MSCs come in contact with HSCs/HSPCs, anchor them and further orient their differentiation and division, which increases the population of stem cells. It is possible that this process is induced by the Cxcl12/CxcR4 signalling axis (36, 53). This is facilitated, as mentioned earlier, by the formation of ‘pockets’ around HSCs/HSPCs, increasing the local concentration of growth factors and signalling molecules, and creating the most productive conditions for the interaction between haematopoietic stem cells and stromal cells (36). Importantly, MSCs were previously described as fibroblastic reticular cells (FRCs), and later as stromal cells or stromal reticular cells (SRCs) also present in the pronephros in fish and, similar to CXCL12^+^ reticular cells, present in mammalian bone marrow (53).

Primitive macrophages and neutrophils, being the cells of the hematopoietic microenvironment, play an important regulatory role in the formation of HSCs/HSPCs in VDA and the migration of these cells to CHT and haematopoietic organs in adult individuals (pronephros, thymus) (2, 19, 23, 47–53). These cells, as mentioned earlier, develop at the early stage of embryonic haematopoiesis, i.e. during the primitive wave of haematopoiesis (16 hpf) (**Figure 1.1.A.a**) and the definitive wave of haematopoiesis (30 - 55 hpf) (**Figure 1.1.A.b**), when HSCs/HSPCs are generated (26-54 hpf). The presence of *mpeg1*^+^ macrophages was demonstrated in VDA and CHT (19, 46, 50). Travnickova et al. (50) reported that primitive macrophages, by releasing Mmp-9 (matrix metalloproteinases 9), induce the degradation of the extracellular matrix (ECM) and mobilize HSCs/HSPCs for migration. However, the actual role of these cells in niches of the early stage of embryonic haematopoiesis is still unclear (19, 37, 50, 67). Primitive neutrophils, like primitive macrophages, express the genes coding matrix metalloproteinases, Mmp-2 and Mmp-9, and these enzymes, apart from the degradation of extracellular matrix proteins, also stimulate the release of HSCs/HSPCs from VDA (Mmp-2) and CHT (Mmp-9) (19, 51). Theodore et al. (51) emphasized that Mmp-2 and Mmp-9 proteins are active at different stages of the formation, development and migration of HSCs/HSPCs and *via* discrete indirect or direct mechanisms, they are involved in processes associated with the signalling inflammatory process, extracellular matrix protein degradation, and the regulation of chemokines activity. In the formation of HSCs/HSPCs, the signalling pathway mediating sterile inflammation (in an environment without the inflammatory/damaging factor) triggered towards endothelial cells in the VDA also plays an important role. This process develops, for example, because of the activity of Tnf-α, a proinflammatory cytokine released mainly by primitive neutrophils (and to a lesser extent by the primitive macrophages), which activates the endothelial cells in VDA. In subsequent events of the process Tnf-α actives specific Tnfr2 (tumour necrosis factor receptor-2), and this increases the expression of Jag1a and activates Notch1a, which may be a receptor present on the surface of HSCs, and ultimately, through the nuclear factor kappa-light-chain-enhancer of activated B cells (NF-kB) active in the formed HSCs, transcription necessary for the release of HSCs/HSPCs from endothelial cells is triggered (67, 74). The formation, release and migration of HSCs/HSPCs is also controlled and promoted by the regulatory activity of Ifn-γ and Ifn type I – the equivalent of IFN-α in mammals (37). The regulatory properties of these cytokines, like Tnf-α, reflect their broad functional potential, which is very important, as it turns out, in the process of embryonic haematopoiesis that, of note, is not related to the inflammatory process/inflammation or protection against infection.

## Haematopoiesis in Adult *Danio Rerio*

In adult fish, haematopoiesis develops in the pronephros. In this organ HSCs/HSPCs are self-renewing and differentiating, giving rise to all lineages of blood cells (19, 28, 36). However, there are still open questions about the site where HSCs are accommodated and differentiate, what the mechanisms regulating the formation of mature morphotic elements of blood in fish and other vertebrates are, and what is the most probable model/paradigm on haematopoiesis. The classical model of haematopoiesis in vertebrates, also in the light of recent studies on *D. rerio* (75), aided with state-of-the art research techniques and regarding “megakaryopoiesis” (18) or the molecular definition of cell populations/clusters in the pronephros and identification of marker genes for cells of specific haematopoietic lineages (75), is still being improved and is a subject of targeted verification (1, 11, 13–15, 18, 76). The proposed models of hematopoiesis and data on this process in vertebrates still raise further doubts and questions, for example about heterogeneity, the presence/localization of HSC/HSPC in the adult body or the definition of a hematopoietic niche, and thus whether the bone marrow in mammals or the anterior kidney in fish, they are the only/final sites of the blood formation process. Today we know that not (1, 14, 15).

**Figure 2:**
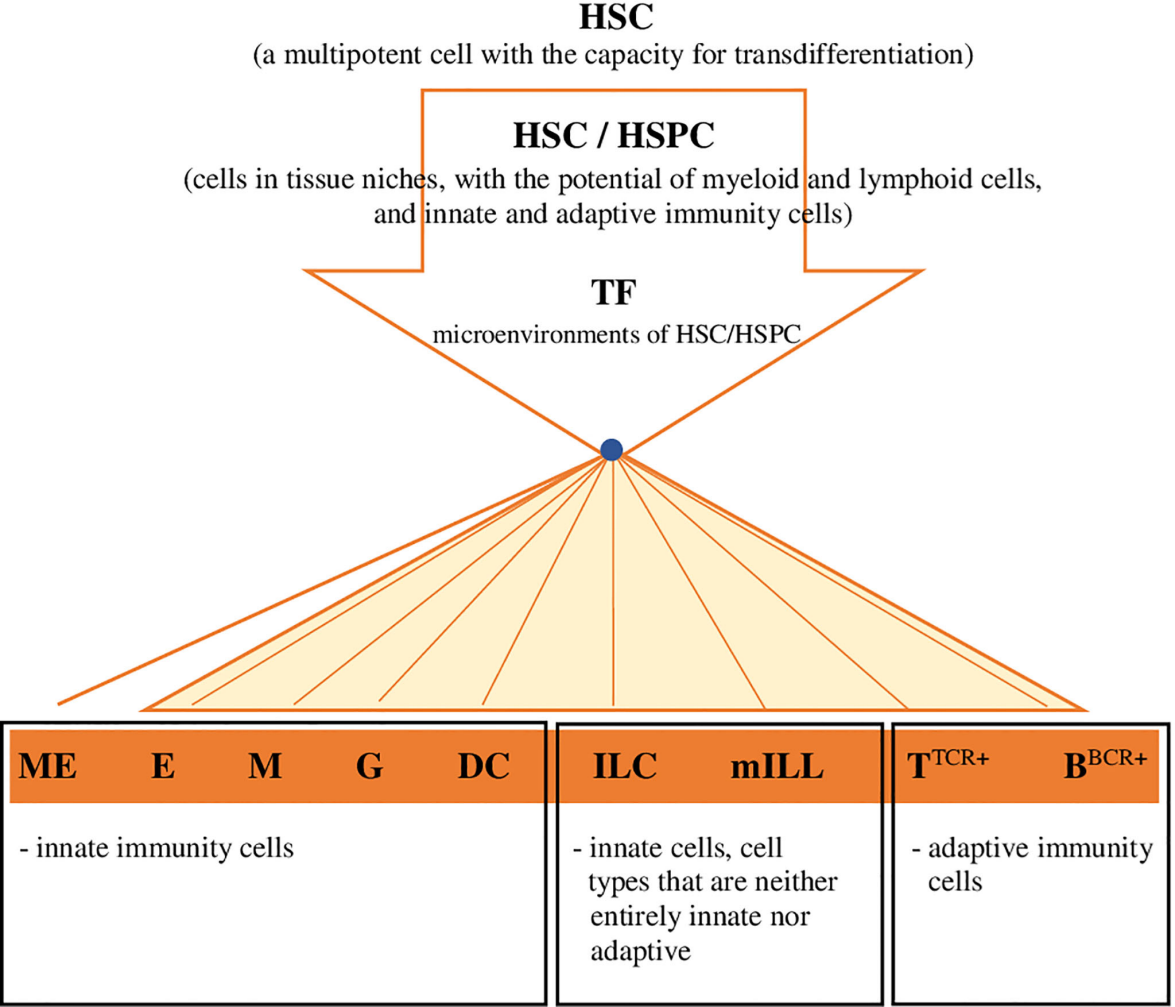
Hypothetical model for the development and differentiation of HSCs, in multiple directions specific for lineages, continuous and without creating a distinct hierarchy of organized progenitor populations (proposed by the authors). Explanatory noted: HSC, haematopoietic stem cell; HSC/HSPC, haematopoietic stem cell/hematopoietic progenitor cell (migratory/tissue,resident); TF, transcription factor (or sequence,specific DNA,binding factor) in the environment of HSC-HSPC cells; ME, megakaryocytes/source of platelets in mammals/thrombocytes in fish; E, erthcrocytes; M, monocytes/macrophages; G, granulocytes; DC, dendritic cells; ILC (ILC1; ILC2; ILC3/ NK; LTi), innate lymphoid cells; mILL (Tγδ lymohocytes [IEL, intraepithelial lymphocytes]; mucosal associated invariant T cells [MAIT]; NKT cells; B1 lymphocytes [unconventional BCD5+ lymphocytes]; marginal zone B cells), mammalian unconventional or innate-like lymphocytes; TTCR+, TTCR+ lymphocytes and their subpopulations; BBCR+, B BCR+ lymphocytes and their subpopulations.

Considering the latest findings on haematopoiesis in vertebrates, including mammals and fish, the conventional concept of HSCs, including their presence, development and differentiation, is evolving into a completely different paradigm. HSCs/HSPCs form a flexible heterogeneous population of cells with different potential for self-renewal and differentiation (14, 18, 77, 78). In mammals these cells in the state of homeostasis circulate and generate peripheral haematopoiesis, respond to differentiating signals and the presence of antigens, and produce myeloid cells, including dendritic cells (DC), in the periphery (14, 79). HSCs and HSPCs have been detected in the lungs and the gut of mammals - humans and mice (14, 80), and it was demonstrated (79) that these cells can migrate to other organs/tissues and return from the periphery to the bone marrow. It should be emphasized that also in the periphery, the integrity of HSCs is protected by the haematopoietic microenvironment, i.e. niches that are heterogeneous by nature (14), because they are formed, for example, by osteoblasts, endothelial cells, mesenchymal stromal cells, nerve cells and megakaryocytes (ME) (14). Moreover, when exposed to microenvironmental factors, HSCs develop and change autonomously and heteronomously (14). Cheng et al. (1), in the presence of data obtained through the use of advanced single-cell ‘omics’ techniques, indicated a model of continuous differentiation of HSCs, which is characterized by the absence of a discernible hierarchy. HSCs develop and differentiate gradually, in many directions, continuously and without creating a distinct hierarchy of organized progenitor populations (**Figure 2**). Cheng et al. (1) assumed that because of the identified heterogeneity of HSCs, the ability of the development of these cells into specific lineages is already acquired before their differentiation. Studies on samples of human bone marrow (11) and in *D*. *rerio* (18) revealed that profiled cells with a restricted monolineage originate from the continuum of undifferentiated HSPCs, i.e. cells that have the potential of myeloid and lymphoid cells, either innate or adaptive (**Figure 2**). A separate haematopoietic lineage was identified among HSCs, ‘preventing’ the formation of megakaryocytes (the equivalent of thrombocytes in teleost fish) and platelets, which acquire properties specific just for that lineage (76). This fact is particularly interesting, also in consideration of the functional potential (haemostatic and immune properties) of these cells in vertebrates (platelets in mammals and thrombocytes in fish) at various stages of phylogeny (5). Brown and Ceredig (15) indicated that it is very likely that in mammals HSCs and their ‘progeny’ are pluripotent, because regardless of the predetermined fate of their development they may switch it to an alternative one, closely related, and during their development/differentiation they are sensitive to many cytokines (THPO - thrombopoietin, EPO - erythropoietin, CSF3 - colony stimulating factor 3 [granulocyte-colony stimulating factor - G-CSF], CSF1 - colony stimulating factor 1 [macrophage-colony stimulating factor - M-CSF], CSF2 - colony stimulating factor 2 [granulocyte-macrophage- colony stimulating factor - GM-CSF], FLT3L - FMS-like tyrosine kinase 3 ligand), which determine the profiling of HSCs and formation of a specific lineage of haemopoietic cells. The local increase in the concentration of certain cytokines and the autoregulated expression of the receptor specific for this cytokine most likely determine the fate of HSCs’ differentiation process, which depends on the effects of cytokine-receptor interaction (15).

In *D. rerio* HSCs/HSPCs migrate from CHT and further colonize the thymus, where T lymphocytes are formed (3 dpf), and the pronephros, where erythroid, myeloid and lymphoid – B lymphocytes cells are formed, except T lymphocytes (4 dpf) (2, 19, 32, 56, 62–64, 81). As emphasized by Macaulay et al. (18), zebrafish have cells of all haematopoietic lineages and orthologs of transcription factors and other genes that are also involved in the process of haematopoiesis in mammals, namely *tal1*, *lmo2*, *lyl1*, *gata2*, *runx1*, *meis1*, *myb* and *erg* specific for HSCs, *fli1*, *gfi1b*, *gata1*, *cd61*, *cd42b* specific for megakaryocyte/erythroid lineages, and *gfi1*, *spi1b* specific for myeloid cell lineage. It should be added that *D*. *rerio* has two GATA2 orthologs, ie *gata2a* and *gata2b*. The dominant and required for maintenance of HSCs is *gata2b*, expressed on HSCs and HSPCs, while *gata2a* dominates in the vascular system, including hemogenic endothelial cells (30). The high degree of comparability/functional similarity between fish and mammals also relates to signalling pathways and transcription mechanisms that are active during haematopoiesis in these two taxonomic groups of vertebrates (18, 23). According to Macaulay et al. (18), adult haematopoiesis in *D*. *rerio* is, as in mammals, continuous and asynchronous/flexible, and the pronephros in fish, like the bone marrow in mammals, accommodates all types of haematopoietic cells at different stages of differentiation. This was shown in studies on the origin and development of thrombocytes (18), based on single cell RNA sequencing (scRNA-seq), which allows for the analysis of differential expression (DE), grouping and classification of cells, but also the reconstruction of cell differentiation trajectory. This study (18) revealed that the differentiation of cells (acquisition of specific phenotypes) progresses along a one-dimensional, non-branching path. It was also found (18) that this process is correlated/consistent with the transcriptional programme, which is reflected in the stimulated or suppressed expression of genes specific for the programme of development, differentiation and functional determination of cells from a particular lineage, or restricting the proliferation of cells and their translational capacity. Macaulay et al. (18) emphasized that as this process continues, the number of expressed genes and mRNA content in the cell is reduced and limited to those which define a specific cell lineage. In addition to these data, information on defined sets of genes specific for HSCs, HSPCs, megakaryocytes/thrombocytes *^runx1^*,*^cd41^* ^(kidney marrow [pronephros])^; neutrophils *^mpx^* ^(kidney marrow [pronephros])^; NK cells *^lck^*^/^*^rag1^*^-/- (kidney marrow [pronephros])^; B cells *^rag2^* ^(kidney marrow [pronephros])^; mature T cells *^lck^* ^(thymus)^ is also important (18). The heterogeneity of cells in the pronephros in adult *D*. *rerio* was also demonstrated by Tang et al. (75), based on the massively parallel transcriptomic method using indexing droplets (InDrops), single-cell RNA sequencing and t-distributed stochastic neighbour embedding (tSNE). In these studies, major haematopoietic cells in the pronephros of zebrafish were defined, i.e. neutrophils, progenitors, erythroid cells, HSCs/thrombocytes, B cells, T/NK cells, myeloid cells, macrophages, as well as seven unique kidney stromal-cell types. Novel genes of specific haematopoietic cell lineages were also identified (HSCs/HSPCs*^runx1^*, thrombocytes*^cd41^*, neutrophils*^mpx^*, T/NK*^lck^* cells, NK*^lck/rag1-/-^* cells, B*^rag2^* cells), and a new population of NKL*^mpeg1.1^*,*^ccl33.3^*,*^nkl.3^*,*^nkl.4,prf1.2,prf1.7^* cells (NK-like), which conserve the genes typical for cells with cytotoxic and lytic capacity despite the lack of their association with T*^lck^* lymphocytes. The analysis of findings (75) suggests that classically defined HSPCs involved in the lineage of erythroids and thrombocytes may also include progenitor cells with closely related transcriptional programmes.

## Concluding Remarks

The presented information shows that haematopoiesis in *D*. *rerio*, as in mammals, is a complex process, both at the embryonic stage and in adult individuals. Findings on the development of haematopoiesis and the role of the microenvironment/niche in the regulation of the formation and function of HSCs/HSPCs shed a new light on this process, especially in the context of details associated with the differentiation of HSCs and the resulting need to modify the paradigm on haematopoiesis. Despite the discoveries made to date, the process of haematopoiesis in *D*. *rerio* and other vertebrates requires further studies to explain, for example, the epigenetic characteristics of cells from specific haematopoietic lineages, taking into account different stages of development and a continuum of differentiation, or the presence of HSCs outside the bone marrow/pronephros niche, and define the ‘local’ capacity of these cells for self-renewal and differentiation into mature forms of certain haematopoietic lineages.

## Author Contributions

All authors contributed to the article and approved the submitted version.

## Conflict of Interest

The authors declare that the research was conducted in the absence of any commercial or financial relationships that could be construed as a potential conflict of interest.

## Publisher’s Note

All claims expressed in this article are solely those of the authors and do not necessarily represent those of their affiliated organizations, or those of the publisher, the editors and the reviewers. Any product that may be evaluated in this article, or claim that may be made by its manufacturer, is not guaranteed or endorsed by the publisher.
